# A novel polycaprolactone/carbon nanofiber composite as a conductive neural guidance channel: an in vitro and in vivo study

**DOI:** 10.1007/s40204-019-00121-3

**Published:** 2019-12-12

**Authors:** Saeed Farzamfar, Majid Salehi, Seyed Mohammad Tavangar, Javad Verdi, Korosh Mansouri, Arman Ai, Ziba Veisi Malekshahi, Jafar Ai

**Affiliations:** 1 Department of Tissue Engineering and Applied Cell Sciences, School of Advanced Technologies in Medicine, Tehran University of Medical Sciences, Tehran, Iran; 2 Department of Tissue Engineering, School of Medicine, Shahroud University of Medical Sciences, Shahroud, Iran; 3 Tissue Engineering and Stem Cells Research Center, Shahroud University of Medical Sciences, Shahroud, Iran; 4 Department of Pathology, Shariati Hospital, Tehran University of Medical Sciences, Tehran, Iran; 5 Neuromusculoskletal Research Centre Firozgar Hospital, Iran University of Medical Sciences, Tehran, Iran; 6 School of Medicine, Tehran University of Medical Sciences, Tehran, 141556447 Iran

**Keywords:** Carbon nanofiber, Polycaprolactone, Electrical conductivity, Sciatic nerve regeneration

## Abstract

The current study aimed to investigate the potential of carbon nanofibers to promote peripheral nerve regeneration. The carbon nanofiber-imbedded scaffolds were produced from polycaprolactone and carbon nanofibers using thermally induced phase separation method. Electrospinning technique was utilized to fabricate polycaprolactone/collagen nanofibrous sheets. The incorporation of carbon nanofibers into polycaprolactone’s matrix significantly reduced its electrical resistance from 4.3 × 10^9^ ± 0.34 × 10^9^ Ω to 8.7 × 10^4^ ± 1.2 × 10^4^ Ω. Further in vitro studies showed that polycaprolactone/carbon nanofiber scaffolds had the porosity of 82.9 ± 3.7% and degradation rate of 1.84 ± 0.37% after 30 days and 3.58 ± 0.39% after 60 days. The fabricated scaffolds were favorable for PC-12 cells attachment and proliferation. Neural guidance channels were produced from the polycaprolactone/carbon nanofiber composites using water jet cutter machine then incorporated with PCL/collagen nanofibrous sheets. The composites were implanted into severed rat sciatic nerve. After 12 weeks, the results of histopathological examinations and functional analysis proved that conductive conduit out-performed the non-conductive type and induced no toxicity or immunogenic reactions, suggesting its potential applicability to treat peripheral nerve damage in the clinic.

## Introduction

Unlike the central nervous system, peripheral nerves have the capacity to regenerate the injured tissue (Faroni et al. 2015). However, in most cases this capacity is disrupted and peripheral nerve repair is incomplete with poor functional recovery (Gu et al. 2014). Currently, the treatment of choice is end-to-end epineurial suturing of the proximal and distal nerve stumps (Siemionow and Brzezicki 2009). In case of critical size defects, where tension-free neurorrhaphy is not possible, autologous nerve grafting is considered as the gold standard (Muheremu and Ao 2015). However, this treatment modality is associated with several shortcomings including the sacrifice of a healthy nerve, need for secondary surgery, and extra damage to donor site (Habre et al. 2018). Collectively, these disadvantages have driven efforts toward developing alternative therapeutic strategies. With the progress of tissue engineering, a variety of natural and/or synthetic polymers have been utilized to produce neural guidance channels (NGCs) in an attempt to substitute autologous nerve grafts (Farzamfar et al. 2018; Nectow et al. 2011). The NGCs preclude the invasion of fibrous tissue into the gap between two stumps. In addition, NGCs can entrap and enrich the neurotrophic factors within the channel and build a permissive environment for nerve regeneration (Muheremu and Ao 2015). Appropriate choice of biomaterial and fabrication method is crucial to design an ideal NGC (Sarker et al. 2018). In this regard, a variety of synthetic and/or natural materials have been tested in neural tissue engineering (Nectow et al. 2011; Yu et al. 2011). Among synthetic biomaterials poly(ε-caprolactone) (PCL) due to its biocompatibility, bioresorbability, and lack of immunogenicity provide a favorable environment in the context of tissue repair and reconstruction (Smith et al. 2015). However, cell affinity towards PCL is generally poor as a result of its lack of cell attachment sites and high hydrophobicity (Lee et al. 2012; Schnell et al. 2007). To improve cell–scaffold interactions, one useful strategy is to incorporate cell-recognition domains such as Arg-Gly-Asp (RGD) groups and extracellular matrix (ECM) bioactive proteins into the PCL scaffolds (Zheng et al. 2012). Collagen is the main component of native ECM. Therefore, the selection of collagen as a scaffolding material is most favored in tissue engineering. However, fast degradation may hamper its application as a platform to guide axonal growth (Glowacki and Mizuno 2008). Therefore, we used a combination of PCL and collagen to develop NGCs. Developing scaffolds with structural similarity to natural ECM at the nanoscale has a significant effect on organization of cells and the corresponding tissue properties (Lannutti et al. 2007). Furthermore, the optimum porosity of the NGCs wall will facilitate metabolites diffusion which is one of the essential requirements of an NGC (Keane and Badylak 2014). To provide such scaffolding structures, the combination of thermally induced phase separation (TIPS) and electrospinning methods can be used. Electrospun fibers have been investigated as promising tissue engineering scaffolds for peripheral nerve regeneration (Prabhakaran et al. 2008; Wang et al. 2008). TIPS method can be utilized to yield porous scaffolds with suitable interconnectivity of the pores (Dong 2015; Salehi et al. 2015). It has been shown that electrically conductive NGCs have superior regenerative capacity than non-conductive NGCs (Salehi et al. 2018). Since PCL and collagen are non-conductive, electrical properties of PCL/collagen composite scaffold can be improved by incorporation of filler materials. In this regard, it has been previously shown that incorporation of carbon nanofibers (CNF) in the scaffolds can endow them with the conduction capability (Stout et al. 2011). Since the effects of CNFs in peripheral nerve regeneration have not been studied yet, the basic aim of the current research was to investigate the regenerative potential of PCL/CNF conduits in a rat model of sciatic nerve defect.

## Methods and materials

The polymers and solvents were obtained from Sigma-Aldrich (St. Louis, USA) and Merck (Darmstadt, Germany), respectively, unless otherwise noted.

### Fabrication of the NGCs through thermally induced phase separation and electrospinning methods

**Fig. 1:**
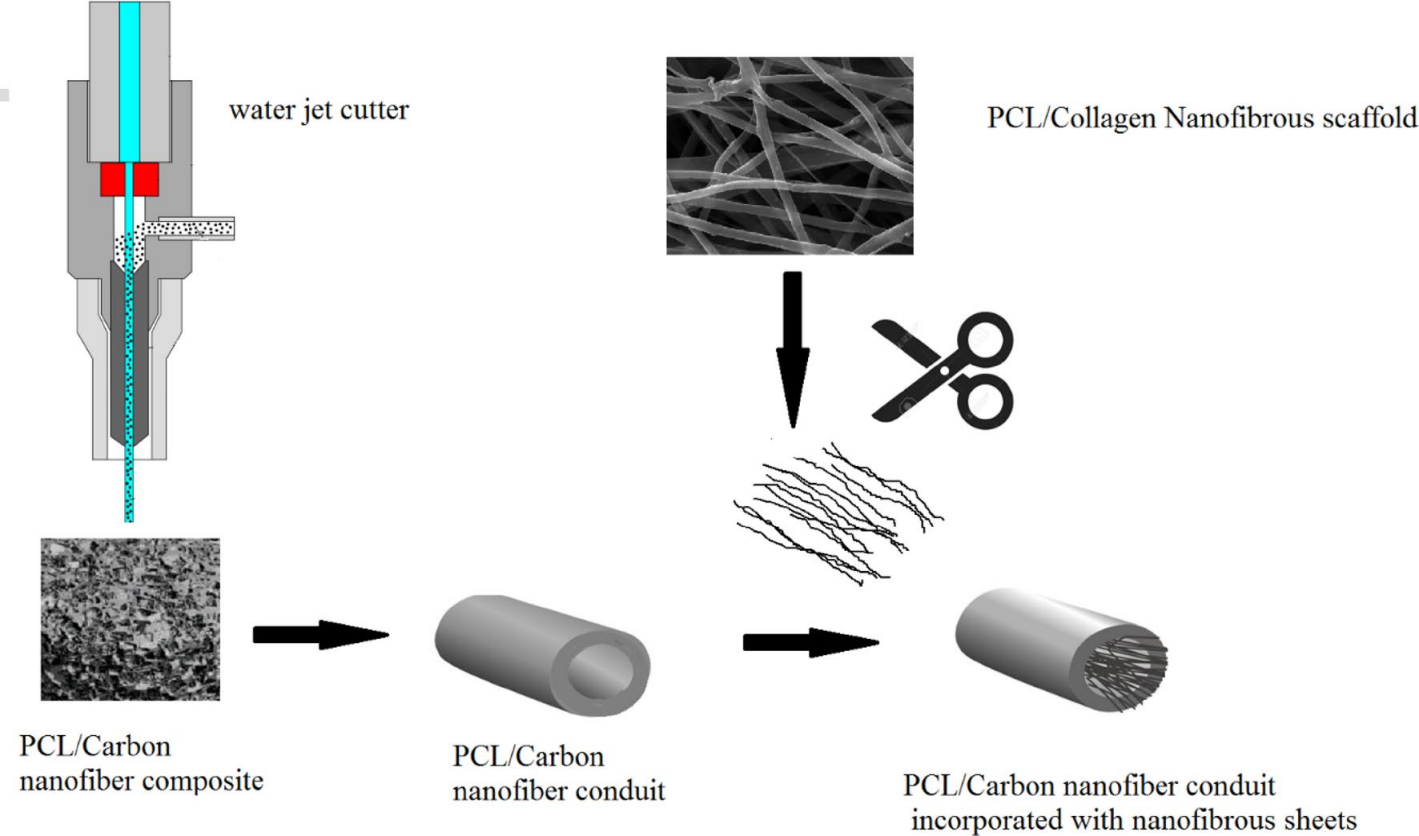
Schematic illustration for the production of the neural guidance channels

Carbon nanofibers (US Research Nanomaterials Inc. Cas no. 308068-56-6) were dispersed in 1,4-dioxane using a magnetic stirrer for 6 h and then ultra-sonicated for 3 h at room temperature. After this time period, polycaprolactone (PCL, *M*_w_ ~ 80.000 kDa) was added to the CNF containing 1,4-dioxane to the final concentration of 6% w/v (CNF weight in the solution was 10% of PCL weight) and stirred for 24 h and then ultra-sonicated to homogenously disperse the CNF in the PCL matrix. The resulting mixture was transferred to − 20 °C and maintained for 4 h. After 4 h, the solidified mixture was transferred to − 80 °C and kept overnight. The sample was freeze-dried (Telstar co., Terrassa, Spain) for 48 h. The fabricated scaffold was shaped into 14-mm conduits using a water jet cutter machine. In order to fabricate PCL/collagen nanofibers, PCL and collagen were separately dissolved in acetic acid at concentrations of 14% (w/v) and 5% (w/v), respectively, and then mixed at the volume ratio of 70:30. The prepared solution was transferred into a 10-mL disposable syringe and fixed in a syringe pump (SP1000, Fanavaran Nano-Meghyas, Iran). An 18 gauge stainless metal needle was placed at the tip of the syringe and the needle tip to collecting mandrel distance was set at 15 cm. Electrospinning was performed by applying a positive high voltage (18 kV) and the polymer was fed at the rate of 0.5 mL/h. Electrospun matrices were cut into thin thread like pieces and put into the NGC’s lumen at the time of implantation. Schematic illustration of the NGC’s fabrication process is shown in Fig. 1.

## Characterization of the scaffolds

### Scanning electron microscopy (SEM) analysis of scaffolds

The microstructure of the produced composite was evaluated by scanning electron microscope (SEM; DSM 960A, Zeiss, Germany) followed by coating with gold for 250 s using a sputter coater (SCD 004, Balzers, Germany).

### Weight loss measurement

In vitro degradation measurement was carried out by immersing the NGCs in 10 mL of PBS at 37 °C in an incubator. After selected times (30 and 60 days), conduits were taken out and freeze-dried. Weight loss was calculated using the following equation:

1 $${\text{Weight loss }}\left( {\text{\% }} \right) = \frac{{W_{0} - W_{1} }}{{W_{0} }} \times 100,$$

where “*W*_0_” indicates the initial weight of NGCs and “*W*_1_” is the dry weight of the NGCs after removing from the PBS solution.

### Porosity measurement

Liquid displacement method and the flowing equation were used to determine the NGC’s porosity:

2 $${\text{Porosity }}\left( \% \right) = \frac{V1 - V3}{V2 - V3} \times 100 ,$$

where *V*_1_ stands for the volume of 96% ethanol before the immersion of NGC, *V*_2_ indicates its volume after NGC immersion and *V*_3_ is the volume of the ethanol after the NGC removal.

### Conductivity measurement

The surface resistance of fabricated scaffolds was evaluated using a conventional four-probe method. The composite scaffolds were cut in 2 × 3 cm^2^ shape and put on the four-probe instrument (Santa Nama Javan Co). By applying a voltage the corresponding electrical current was recorded. The surface resistance of the samples was determined by using the following equation:

3 $$R_{\text{s}} \left( \varOmega \right) = \frac{\pi }{\ln \left( 2 \right)}\frac{V}{I}{\text{DMSO,}}$$

where *R*_s_ indicates the surface resistance, *V* represents voltage and *I* shows the current passed through the probes.

### Cell viability assay

3-(4,5-Dimethylthiazol-2-yl)-2,5-diphenyltetrazolium bromide (MTT) assay was used to determine the viability of PC-12 cells seeded on the PCL/CNF scaffolds after 1 and 3 days of cell seeding. Scaffolds were placed in each well of 96-well plates and 1 × 10^4^ of PC-12 cells were seeded onto each sample. The culture medium on the samples was replaced with 100 µL of 0.5 mg/mL MTT solution and incubated for 4 h. After this time, the MTT solution was aspirated and 100 µL of dimethyl sulfoxide (DMSO) solution was added to dissolve the formazan crystals and kept at dark on a rotary shaker for 10 min. The solution was transferred to new well and the absorbance values of the samples were read at 570 nm.

### Cell adhesion studies

A number of 7 × 10 ^3^ PC-12 cells were cultured on the electrospun scaffolds for 3 days. After this time period, the samples were washed with PBS and then fixed in 2.5% glutaraldehyde solution at room temperature for 2 h. The cell–scaffold constructs were then dehydrated using graded concentrations of ethanol in distilled water. The samples were freeze-dried and after sputter coating imaged under the SEM.

## In vivo studies

### Sciatic nerve defect

Male Wistar rats (3 months old, weighing 250–270 g) were purchased from Pasteur Institute (Tehran, Iran). Animal experiments were approved by the Ethics Committee of Tehran University of Medical Sciences and were carried out in accordance with the University guidelines. The animals were divided into 5 groups: (1) positive control (rats without sciatic nerve injury); (2) negative control (with sciatic nerve injury but without treatment); (3) autograft; (4) PCL/CNF group; (5) PCL group. To induce anesthesia, a mixture of ketamine 5% and xylazine 2% (70 mg ketamine and 6 mg xylazine/1000 g body weight of animals) was administered by intraperitoneal injection. The right thigh of the rats was then shaved and disinfected by the povidone–iodine solution. A skin incision was made and the sciatic nerve was exposed. The sciatic nerve was severed in the middle and a 10-mm-long segment was resected. Proximal and distal nerve stumps were inserted into the NGCs leaving a 10-mm gap between two ends and the nerve tissue was secured in place by a 6-0 nylon suture. In the autograft group resected nerve was reversed and sutured back to the proximal and distal nerve ends.

### Walking-foot-print analysis

Sciatic functional index (SFI) was investigated at 4th, 8th, and 12th week post-surgery. An acrylic corridor with the size of 43 cm length, 8.70 cm width and 5.50 cm height ended to a darkened goal box was prepared and its floor was covered by white papers. At specific time points, rat’s hind paws were dipped into ink and allowed to walk through the corridor, and their foot prints were recorded on the papers. The SFI values for each group were calculated according to our previous study (Farzamfar et al. 2018). SFI = 0 indicates the normal function, while the SFI = − 100 represented the complete loss of function.

### Functional assessment of sensory recovery (hot plate test)

The rat’s sensory function recovery was investigated at 12 weeks post-surgery. Animals were placed on a hot plate (56 °C) and the cut-off time was set at 12 s. The time passed on the hot plate until the onset of rat’s reaction (by licking the paws or jumping) was recorded as hot plate latency time (HPLT).

### Nerve conduction test

Twelve weeks after surgery, the animals were anesthetized by intraperitoneal injection of ketamine 70 mg/xylazine 6 mg/kg body weight) and the operated limb’s sciatic nerve was exposed and stimulated just before the injury site with an electric stimulus (3–5 mA) using a needle electrode. Cap electrodes were placed on the gastrocnemius muscle (filtering frequency of 10 Hz to 10 kHz, the sensitivity of 2 mV/division and sweep speed of 1 ms/division) and the compound muscle action potential (CMAP) amplitude was measured using an electromyographic recorder.

### Gastrocnemius muscle wet weight loss

After 12 weeks of surgery, rats were killed and the posterior gastrocnemius muscles at both operated and no-operated limbs were harvested and immediately weighed for the calculation of weight loss percentage by using the following equation:

4 $${\text{Gastrocnemius muscle wet weight loss }}\left( \% \right) = \left( { 1- \frac{\text{Wet weight of the muscle on the injured side}}{{ {\text{Wet weight of the muscle on the uninjured side}}}}} \right) \times 100.$$

### Histopathological examination

At the end of 12th-week post-surgery, the animals were killed with an overdose of ketamine (150 mg/kg ketamine, 20 mg/kg xylazine) and their sciatic nerve and gastrocnemius muscle were harvested and fixed in a 10% buffered formalin. After processing and embedding in paraffin, they were sectioned and stained with hematoxylin–eosin (H&E). The prepared samples were imaged under a light microscope (Carl Zeiss, Thornwood) with a digital camera.

### Statistical analysis

The results were statistically analyzed by GraphPad prism (version 5) using one-way ANOVA and the data were expressed as mean ± standard deviation (SD). In all evaluations, *P *< 0.05 was considered as statistically significant.

## Results

### Morphology of the scaffolds

**Fig. 2:**
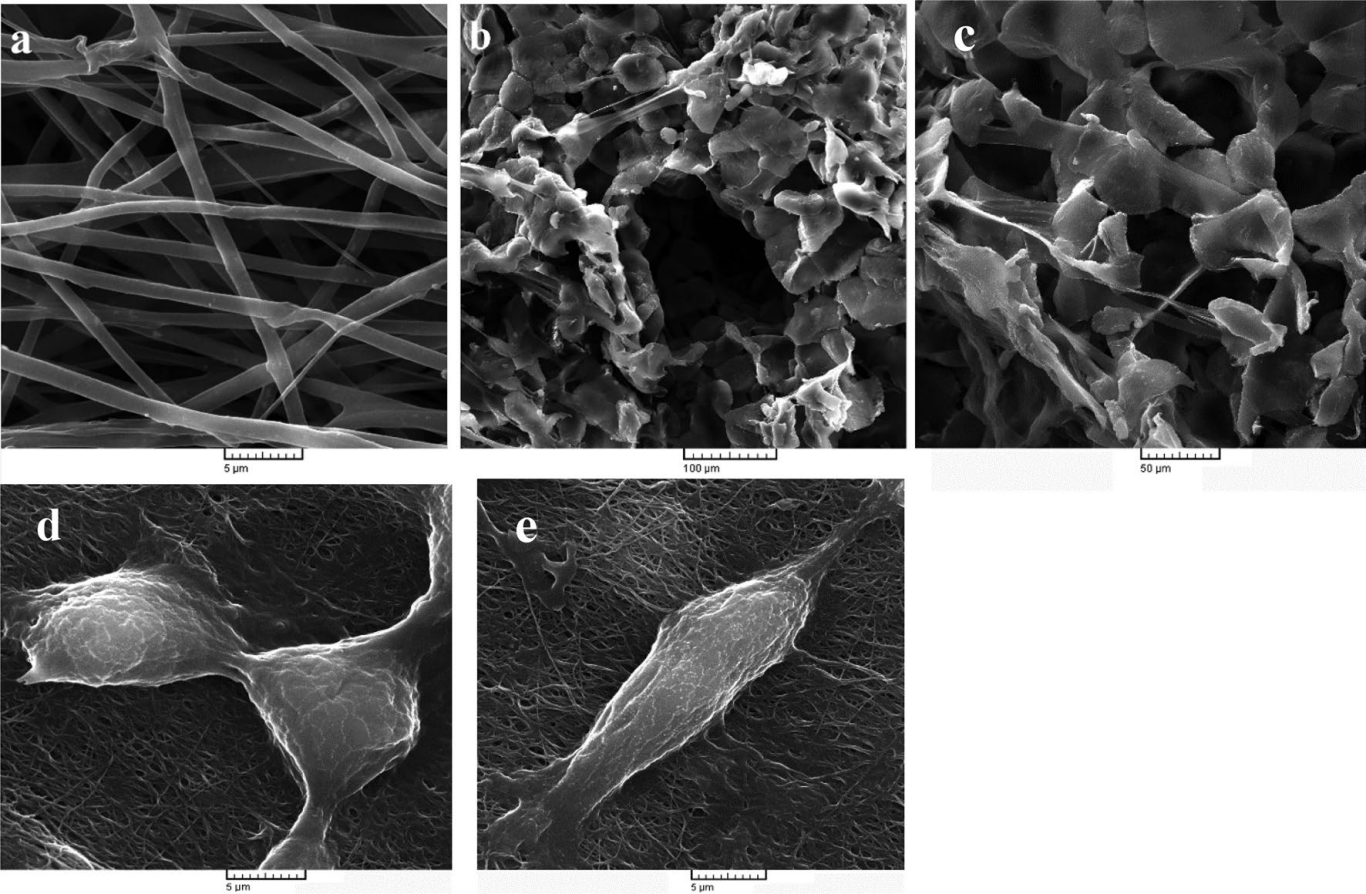
**a** Representative SEM images of the PCL/collagen electrospun matrices; **b**, **c** SEM images of freeze-dried PCL/CNF composite scaffolds, and **e**, **d** SEM images of PC-12 cells cultured on electrospun PCL/collagen nanofibrous scaffolds

The SEM image of PCL/collagen nanofibers (Fig. 2a) illustrated that the fibers had a uniform and smooth morphology with random distribution. The fibrils’ average diameter was measured using Image J (National Institutes of Health, Bethesda, USA) by selecting a total of 30 random points per image. Statistical analysis showed that the fibers had an average diameter of 1234.48 ± 57.3 nm. Electrospun scaffolds have remarkable effects in terms of cellular behavior such as cell orientation and differentiation. Neural cells migrate and deposit ECM proteins on nanofibrous matrices which have been shown to be beneficial for nerve repair (Cao et al. 2009; Nisbet et al. 2009; Yang et al. 2005). Figure 2b, c depicts the porous microstructure of PCL/CNF substrates. The obtained scaffolds had pore size around 100 µm with suitable interconnectivity of the pores.

### Porosity measurement

In order to increase the porosity of the NGCs, they were fabricated using the TIPS method. The high porosity facilitates metabolites diffusion into the NGCs which can provide a permissive environment for nerve repair following damage (de Ruiter et al. 2009). As shown in Table 1, incorporation of CNFs had a negligible effect on average porosity percentage of PCL scaffolds (88.5 ± 5.8% vs. 82.9 ± 3.7% for PCL and PCL/CNF scaffolds, respectively). It is accepted that porosity above 80% is ideal for tissue-engineered scaffolds (Salehi et al. 2018). Therefore, the fabrication technique utilized in this study could produce constructs with suitable porosity.

**Table 1:** Characterization of the scaffolds. Values represent the mean ± SD, *n* = 3, *P < 0.05, **P < 0.01 and ***P < 0.005

Samples	Electrical resistance (Ω)	Porosity (%)	Weight loss after 30 days (%)	Weight loss after 60 days (%)
**PCL**	4.3 × 10^9^ ± .34 × 10^9^	88.5 ± 5.8	2.17 ± .54	3.32 ± .41
**PCL/CNF**	^***^8.7 × 10^4^ ± 1.2 × 10^4^	82.9 ± 3.7	1.84 ± .37	3.58 ± .39

### Degradation rate measurement

Ideal NGCs should be biodegradable to obviate the need for extra surgery for conduit removal after regeneration (de Ruiter et al. 2009). The results of in vitro degradation measurement (Table 1) showed that the PCL and PCL/CNF scaffolds had a minimal weight loss percentage during 60 days. The average weight loss percentage between PCL and PCL/CNF groups was not statistically significant at both time intervals. This value for PCL/CNF scaffolds was 1.84 ± 0.37% and 3.58 ± 0.39% at 30th and 60th day, respectively. While PCL only scaffolds could degrade about 2.17 ± 0.54% and 3.32 ± 0.41% at 30th and 60th day, respectively. The ideal NGC should remain intact during the regeneration time and then should degrade gradually. High degradation rate may lead to swelling and blockade of the tunnel before the re-innervation of distal nerve stump (Muheremu and Ao 2015).

### Cell viability studies

**Fig. 3:**
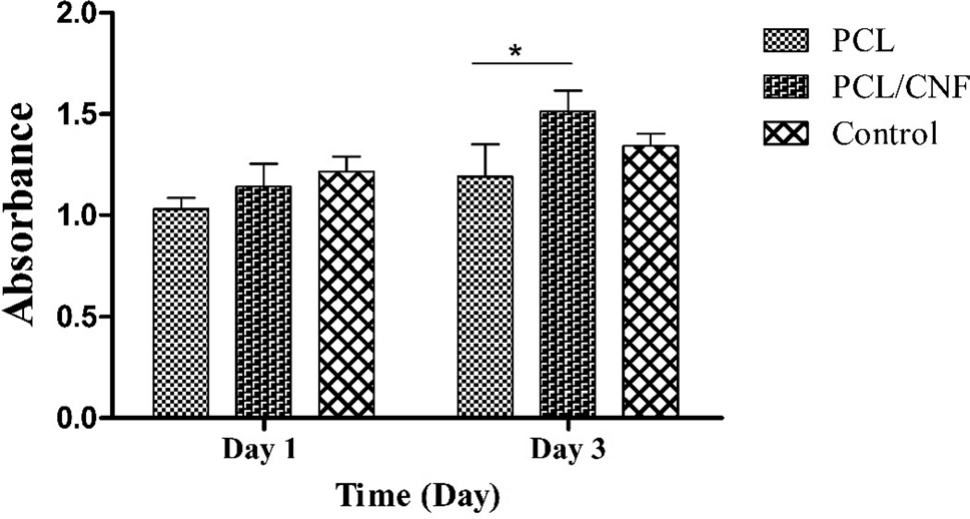
Histogram comparing the viability of PC-12 cells cultured on the PCL and PCL/CNF scaffolds obtained by MTT assay 1 and 3 days after cell seeding. Values represent the mean ± SD, *n* = 3, *P < 0.05, **P < 0.01 and ***P < 0.005

MTT assay was performed to evaluate the viability and proliferation of PC-12 cells on each scaffold. As shown in Fig. 3, at day 1 the activity of PC-12 cells grown on PCL and PCL/CNF scaffolds was lower than control group. However, the difference was not statistically significant. Cells seeded on PCL/CNF substrates demonstrated a significantly higher rate of cell proliferation compared to PCL only scaffolds at day 3. Comparing the absorbance values of the control, PCL, and PCL/CNF groups showed that PCL and PCL/CNF scaffolds had no significant toxicity toward seeded cells. This is in accordance with previous studies which have reported proper cytocompatibility of CNF and PCL (Mirzaei et al. 2016; Naseri-Nosar et al. 2017).

### Cell attachment studies

At the initial stage of cell growth on tissue engineering products, cell attachment which is highly dependent on surface chemistry governs tissue engineering progress (Dong et al. 2015). Scanning electron microscopy was utilized to assess the attachment of PC-12 cells on electrospun PCL/collagen scaffolds (Fig. 2d, e). From these figures, it is possible to observe cells grown on nanofibrous substrate which is well attached and spread out. It is also observable that cells have secreted ECM molecules.

### Electroconductivity measurement

Electroactivity study revealed that incorporation of CNF into PCL matrix significantly reduced its electrical resistance from 4.3 × 10^9^ ± 0.34 × 10^9^ Ω to 8.7 × 10^4^ ± 1.2 × 10^4^ Ω. The homogenously dispersed CNFs form percolated pathway required for an efficient electrical conductivity. The pathway helps the charge transports by hopping from one conductive site to the next and cause the composite to be electrically conductive (Salehi et al. 2018). Electrically conductive conduits can mimic the inherently conductive nature of the nerve tissues and therefore promote the regeneration of peripheral nerve injuries (Anderson et al. 2015).

## In vivo regenerative potential of NGCs

### Sciatic function index (SFI)

**Fig. 4:**
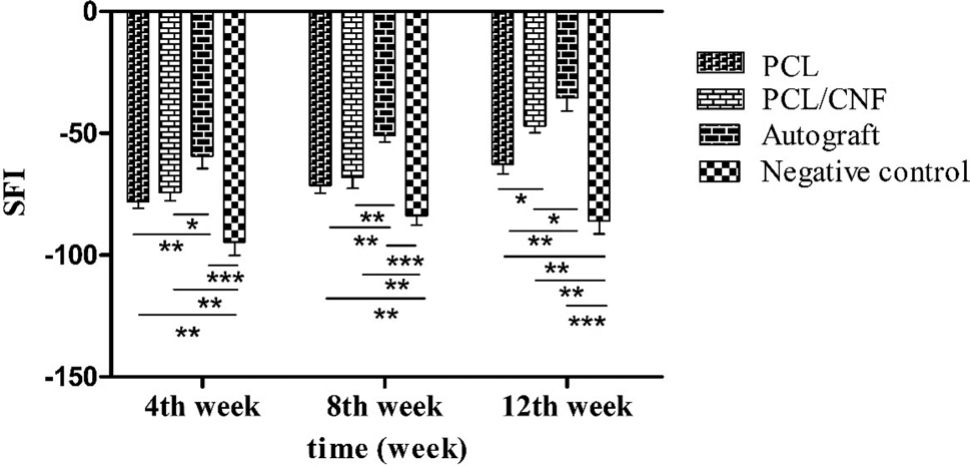
Histogram comparing sciatic functional index study results of different groups 4, 8, and 12 weeks post-surgery. Values represent the mean ± SD, *n* = 3, *P < 0.05, **P < 0.01 and ***P < 0.005

In animal models of sciatic nerve regeneration, SFI is widely used to evaluate the extent of motor function recovery (Lee et al. 2013). Figure 4 illustrates the measured SFI values for all groups. As shown the SFI for all groups had an increasing trend during the study period. Autograft group had significantly greater SFI compared to other groups at all time intervals (*p* value < 0.05) with the values of − 59.33 ± 5.13, − 51.32 ± 2.64, and − 35.66 ± 3.21 at the end of 4th, 8th, and 12th-week post-surgery, respectively. PCL/CNF group had the SFI results of − 74.33 ± 3.19 at the end of 4th week. Its SFI reached the values of -68 ± 4.52 and -45.66 ± 3.05 at the end of 8th and 12th week, respectively. The SFI values for PCL group were − 78.27 ± 2.64, − 71.33 ± 4.21, and − 62.66 ± 4.01 after 4, 8, and 12 weeks, respectively. PCL group had significantly lower SFI values compared to PCL/CNF at 12th-week post-surgery. SFI for the negative control group was close to − 100 at all time intervals, implying complete neuromuscular degeneration.

### Hot plate latency time analysis

**Fig. 5:**
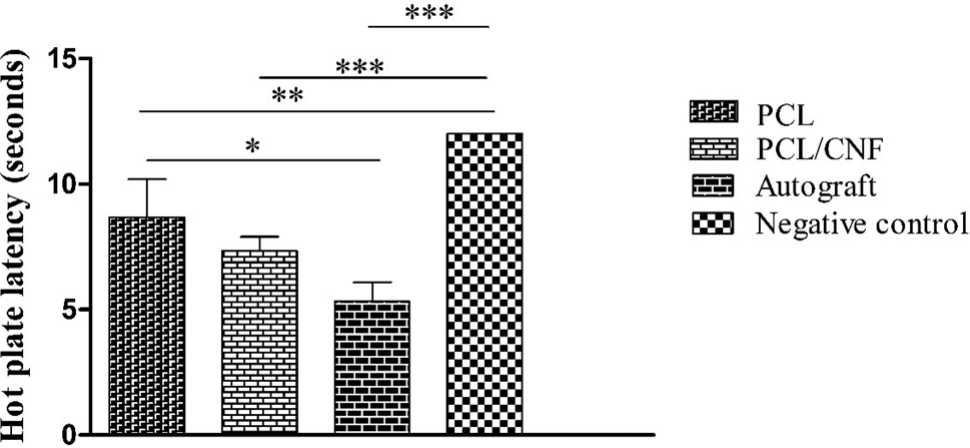
Hot plate latency time measurement results 12 weeks post-surgery. Values represent the mean ± SD, *n* = 3, *P < 0.05, **P < 0.01 and ***P < 0.005

The rats were monitored for their sensitivity to pain by hot plate latency test which reflects the degree of sensory function recovery. As shown in Fig. 5, the autograft group demonstrated a significantly (*p* value < 0.05) better sensory recovery among all groups. However, the normal value of 4 s was not obtained (Hu et al. 1997). Although the animals in the PCL/CNF had a lower hot plate latency time at the end of the 12th-week post-surgery compared with PCL group, the difference was not statistically significant.

### Electrophysiological studies

**Fig. 6:**
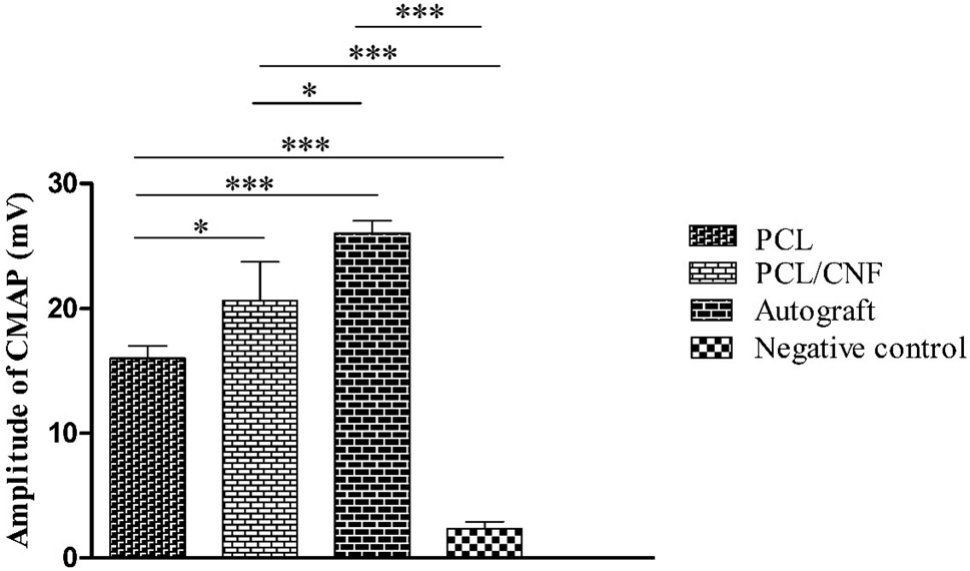
Histogram comparing the amplitudes of compound muscle action potential 12 weeks after surgery. Values demonstrate the mean ± SD, *n* = 3, *P < 0.05, **P < 0.01 and ***P < 0.005

Compound muscle action potential (CMAP) indirectly indicates the motor nerves regeneration (Salehi et al. 2018). Measurements of this value through electrophysiological studies (Fig. 6) showed that severed nerves bridged with autograft nerve had the highest CMAP amplitude. These values for nerves implanted with PCL/CNF scaffolds were found to be significantly higher than that of the rats implanted with PCL conduits indicating higher myelin deposition in PCL/CNF group.

### Gastrocnemius muscle wet weight loss

**Fig. 7:**
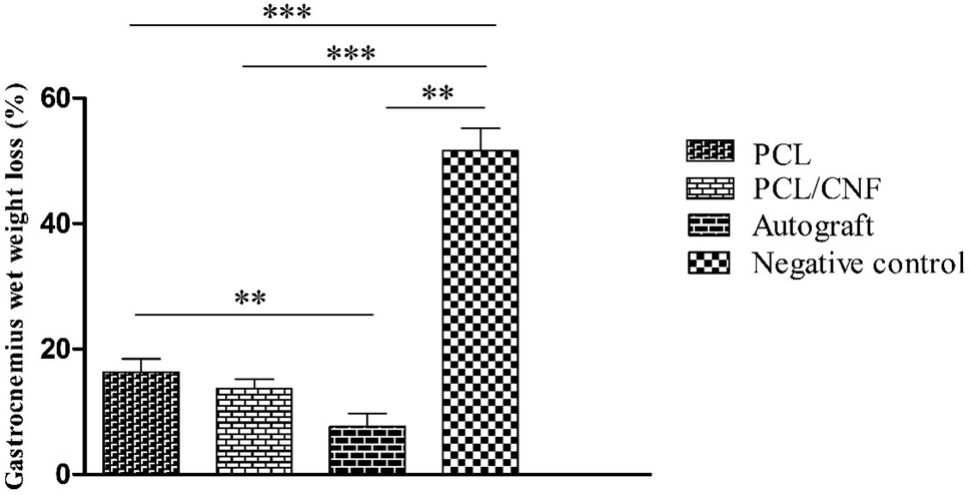
Gastrocnemius muscle wet weight loss percentages of different groups after 12 weeks of surgery. Values represent the mean ± SD, *n* = 3, *P < 0.05, **P < 0.01 and ***P < 0.005

Sciatic nerve injury results in atrophy and weight loss of gastrocnemius muscle. The wet weight loss of the gastrocnemius muscle can indirectly reflect the efficacy of muscle re-innervation (Farzamfar et al. 2018). Figure 7 illustrates that the PCL/CNF group had a lower wet weight loss percentage of this muscle compared to PCL group. However, the difference was not statistically significant (13.66 ± 1.52% vs. 16.33 ± 2.08, *p* value < 0.05). Table 2 summarizes the results of functional analysis tests.

**Table 2:** Summary of functional analysis results

	Sciatic function index (12th week)	Hot plate latency time	Compound muscle action potential	Gastrocnemius muscle wet weight loss
PCL	− 62.66 ± 4.01	8.66 ± 1.52	16.78 ± 1.94	16.33 ± 2.08
PCL/CNF	− 45.66 ± 3.05	7.33 ± .57	20.66 ± 3.05	13.66 ± 1.52
Autograft	− 35.66 ± 3.21	5.33 ± .76	26.45 ± 2.54	8.42 ± 2.41
Negative control	− 86.00 ± 5.29	12	2.33 ± .57	51.73 ± 3.60

### Histopathological assessments

**Fig. 8:**
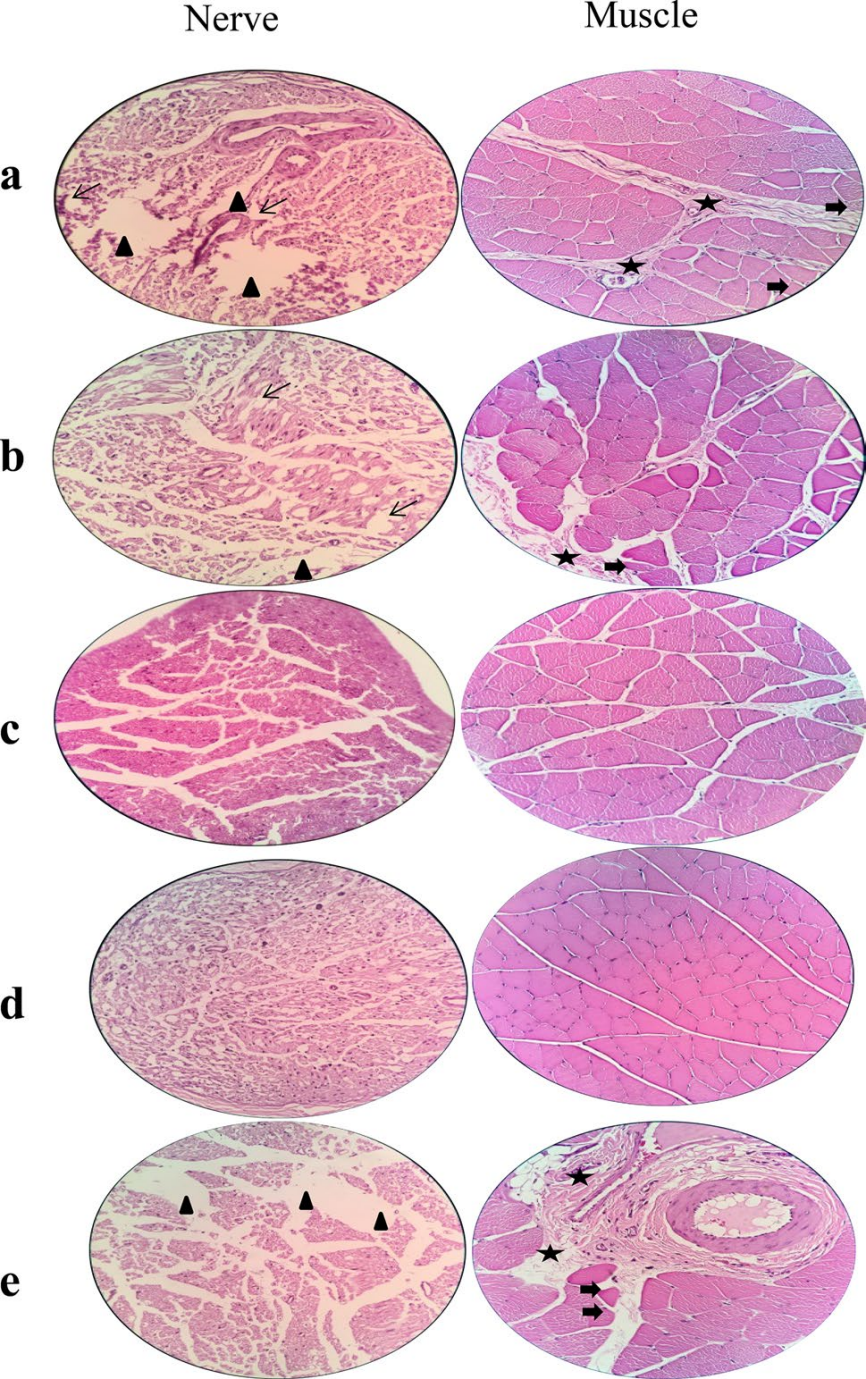
Histopathological images of the sciatic nerve and gastrocnemius muscle (× 400) cross-sections stained by hematoxylin–eosin (H&E) at the end of 12th-week post-surgery. **a** PCL conduit, **b** PCL/CNF conduit, **c** autograft, **d** positive control, **e** negative control. Narrow arrows: focal epineurium loss, asterisks: collagen hyperplasia, arrowheads: vacuolation, thickened arrows: atrophied muscle fiber

After 12 weeks the animals were humanely killed and their sciatic nerve and gastrocnemius muscle tissues were removed for histopathological analysis. Then the gastrocnemius muscle was harvested and kept in 10% neutral buffered formalin (NBF, PH. 7.26) for 48 h. No signs of hematoma or infection were seen at the surgery site, indicating good tissue compatibility of the conduits which is in accordance with the MTT study. Figure 8 illustrates the histopathological examination of the sciatic nerves and gastrocnemius muscle of all groups. Microscopical analysis of the nerves in the positive control group demonstrated well-arranged myelinated nerves with no sign of histopathological alterations. In the negative control group, the nerve fibers were seriously damaged that was evidenced by fibrosis, swollen or degenerated nerve fibers accompanied by edema and high level of vacuolation. The histopathological evaluation of the PCL conduit group exhibited a various degree of vacuolation and mild edema. In the PCL/CNF conduit group, the nerve fibers were well-arranged and fibrosis or inflammatory cell infiltration were not seen in this sample. Histopathological study of the gastrocnemius muscle showed that in the positive control group, muscle fibers were intact indicating no pathological changes. On the other hand, the muscle fibers in the negative control group were contracted and degenerated. Furthermore, the myocytes striation was absent, and fibrosis was significantly increased between myocytes and the whole structure was totally damaged. Histopathological findings in the PCL/CNF conduit group demonstrated more like the autograft group. However, some degrees of fibrosis and muscular atrophy were seen. Muscular atrophy and various degrees of fibrosis and muscular shrinkage were observed in the PCL conduit group.

## Discussion

In order to achieve a satisfactory motor and sensory recovery of an injured peripheral nerve, strategies seek to develop a substrate which exploits several cues to modulate neural cells behavior, whether it be to aid regenerating axons to reconnect with nerve distal stump or to encourage growth factors production and axonal sprouting (Gu et al. 2014). Electrical cues delivered through a conductive scaffold play a determining role in peripheral nerve repair following injury (Evans 2001). Several synthetic and/or natural materials have been tested for their applicability in neural tissue engineering (Daly et al. 2011). Among various candidates, CNFs possess unique mechanical and electrical properties that present new opportunities in the field of peripheral nerve tissue regeneration (Ku et al. 2013). In the current study, we examined the healing potential of CNF in the PCL matrix by comparing the regenerative capability of CNF containing and CNF free nerve guides in animal experiments. Overall, functional analysis and histopathological examinations showed that PCL/CNF nerve conduit enhanced nerve regeneration and mitigated muscle atrophy in a higher extent to what was achieved with CNF free NGCs. The higher healing activity of PCL/CNF conduits can be attributed to the inherent electrical conductivity of CNF. We suppose that higher regenerative performance of CNF containing conduits can be due to the fact that CNFs released from the scaffold changed the microenvironment in the NGCs lumen in a manner that was favorable for nerve regeneration. CNFs may transmit self-produced electrical cues between cells and the two ends of the severed sciatic nerve and improve cellular propagation and nerve regeneration (Ghasemi-Mobarakeh et al. 2011). This improved function of nerve tissues could be due to the better ultra-structural organization in the presence of electric fields. It is likely that neurons’ surface receptors are rearranged in response to the presence of the field and signaling through voltage-gated calcium channels trigger ubiquitous second messengers such as cAMP and alter gene expression profile affecting survival and growth (Lanza et al. 2011). Previous studies have confirmed a positive interaction between CNF and neural cells. For example, Stout et al. speculated that higher neural cells densities on PLGA/CNF composites may be as a result of enhanced vitronectin and laminin deposition on CNFs, which in turn will trigger cell attachment and proliferation (Stout et al. 2011). Mirzaei et al. could successfully differentiate human endometrial stem cells into neuron-like cells on random and aligned carbon nanofibers (Mirzaei et al. 2016). Furthermore, previous studies have confirmed the higher healing potential of conductive NGCs compared to non-conductive ones. In this regard, we previously reported that multi-walled carbon nanotubes could significantly improve the electrical activity of polylactic acid nerve guides. In vivo studies revealed that carbon nanotube-containing group resulted in a higher nerve regeneration compared to the same conduit but without carbon nanostructures (Salehi et al. 2018). Das et al. tested the efficacy of a silk-based gold–nanocomposite NGC for peripheral nerve tissue engineering. Conduction measurement showed a 25 times decrease in silk nanofibers resistance when gold nanoparticles were incorporated into the silk matrix. Animal experiments proved the superior healing potential of gold nanoparticle containing conduits (Das et al. 2015). At this time the molecular mechanism involved in CNF’s role in nerve regeneration is not clear, but this may be due to the aforementioned altered microenvironment and/or electrical activity, requiring further studies. With consideration of in vitro and in vivo results presented in this study, PCL/CNF conduits can be applied to treat peripheral nerve defects in clinic; however, adequate follow-ups regarding potential long-term adverse effects of CNFs should be taken into account.

## Conclusion

In the current study, electrically conductive PCL/CNF conduits supported successful nerve regeneration in critical size sciatic nerve defect in the rat model. Further investigations need to be done to monitor long-term possible adverse effects of CNF application to treat nerve defects in the clinic. This preliminary research suggests the potential applicability of CNF in peripheral nerve tissue engineering.
